# Time Series Analysis in Forecasting Mental Addition and Summation Performance

**DOI:** 10.3389/fpsyg.2020.00911

**Published:** 2020-05-20

**Authors:** Anmar Abdul-Rahman

**Affiliations:** Department of Ophthalmology, Counties Manukau DHB, Auckland, New Zealand

**Keywords:** ARIMA model, time series, mathematics, forecasting–methodology, cognitive performance

## Abstract

An ideal performance evaluation metric would be predictive, objective, easy to administer, estimate the variance in performance, and provide a confidence interval for the level of uncertainty. Time series forecasting may provide objective metrics for predictive performance in mental arithmetic. Addition and summation (addition combined with subtraction) using the Japanese Soroban computation system was undertaken over 60 days. The median calculation time in seconds for adding 10 sequential six digit numbers [CT_Add_) was 63 s (interquartile range (IQR) = 12, range 48–127 s], while that for summation (CT_Sum_) was 70 s (IQR = 14, range 53–108 s), and the difference between these times was statistically significant *p* < 0.0001. Using the mean absolute percentage error (MAPE) to measure forecast accuracy, the autoregressive integrated moving average (ARIMA) model predicted a further reduction in both CT_Add_ to a mean of 51.51 ± 13.21 s (AIC = 5403.13) with an error of 6.32%, and CT_Sum_ to a mean of 54.57 ± 15.37 s (AIC = 3852.61) with an error of 8.02% over an additional 100 forecasted trials. When the testing was repeated, the actual mean performance differed by 1.35 and 4.41 s for each of the tasks, respectively, from the ARIMA point forecast value. There was no difference between the ARIMA model and actual performance values (*p*-value CT_Add_ = 1.0, CT_Sum_=0.054). This is in contrast to both Wright's model and linear regression (*p*-value < 0.0001). By accounting for both variability in performance over time and task difficulty, forecasting mental arithmetic performance may be possible using an ARIMA model, with an accuracy exceeding that of both Wright's model and univariate linear regression.

## 1. Introduction

Learning curves aim to model the gain in efficiency (increase in productivity, decrease in activity time, or both) of a repetitive task with increasing experience. The mathematical representation of the learning process is of particular interest across several disciplines including psychology (Mazur and Hastie, 1978; Balkenius and Morén, 1998; Glautier, 2013), medicine (Sutton et al., 1998; Ramsay et al., 2000; Dinçler et al., 2003; Hopper et al., 2007; Harrysson et al., 2014; Blehar et al., 2015), economics/industry (Cunningham, 1980; Lieberman, 1984; Badiru, 1991; Smunt and Watts, 2003) and more recently, artificial intelligence (Schmajuk and Zanutto, 1997; Perlich et al., 2003; Li et al., 2015).

Learning occurs most rapidly early in training, with equal increments in performance requiring a longer practice time in the later stages of the learning process. The classical understanding is that these diminishing returns result in learning curves that are smooth, decelerating functions (Mazur and Hastie, 1978; Jaber and Maurice, 2016). In 1880 Hermann Ebbinghaus first described the learning curve as a forgetting function; in a series of rigorous experiments he approximated the parameter as a negative exponential equation (Murre and Dros, 2015). In 1936 TP Wright investigated direct labor costs of assembling a particular aircraft and noted that the cost decreased with worker experience, a theory subsequently confirmed by other aircraft manufacturers (Wright, 1936). Analogous to Ebbinghaus's forgetting curve, he predicted the acquisition of skill followed a negative power function currently referred to as Wright's Model:

(1) $$\begin{array}{l} y_{t}=a\cdot x^{b} \end{array}$$

Where *y*_*t*_ = the cumulative average time per unit, x = the cumulative number of units produced, a = the time to produce the first unit and b = learning coefficient (the slope of the function) ranging from −1 to 0; values close to −1 indicate a high learning rate and fast adaptation to task execution. Subsequently, JR Crawford described an incremental unit time model aimed at improving time representation in the algorithm, by substituting (x) in Wrights' model with the algebraic midpoint of the time required to produce a batch of units; this modification was a consequence of an observation that the time to complete a task decreased by a constant percentage, whenever the sum of the units doubled (Crawford, 1944; Jones, 2018). Three-parameter, two-parameter and the constant time exponential models were described to improve longterm predictions (Knecht, 1974; Mazur and Hastie, 1978). These algorithms were outperformed by multi-parameter hyperbolic models, where neutral, positive and negative learning episodes are represented through corresponding variable slope smooth curve profiles (Mazur and Hastie, 1978; Nembhard and Uzumeri, 2000; Shafer et al., 2001; Anzanello and Fogliatto, 2007). While the conventional univariate learning curves express a quantitative dependent variable in terms of an independent variable, multivariate models were eventually formulated to encode both qualitative and quantitative factors influencing the learning process (Badiru, 1992).

The smooth curves generated by these formal models provide an estimate at the average level of a set of observations. However, variation in performance demands a more rigorous representation of the learning process. This variation can be represented in a time series through two stochastic terms. an autoregressive term (AR), calculated as a weighted value from another point in the series, and a moving average (MA), which is estimated from the error terms in the series (Hyndman and Athanasopoulos, 2018). Characterization of time series using either an AR, MA, or combined ARMA processes was suggested independently in the 1920s by the Russian statistician and economist Eugen Slutsky (Slutzky, 1937), and the British statistician George Yule (Yule, 1921, 1926, 1927). It was not until the 1970s when Box and Jenkins described the autoregressive integrated moving average (ARIMA) model, which uses differencing of successive observations to render the series stationary, which is an essential property of the series for statistical validity (Milgate and Newman, 1990; Manuca and Savit, 1996). This study aims to investigate whether accounting for variance in the mental arithmetic using an ARIMA model is more accurate at forecasting performance, compared to Wright's model and univariate linear regression.

## 2. Materials and Methods

### 2.1. Test Description

**Figure 1:**
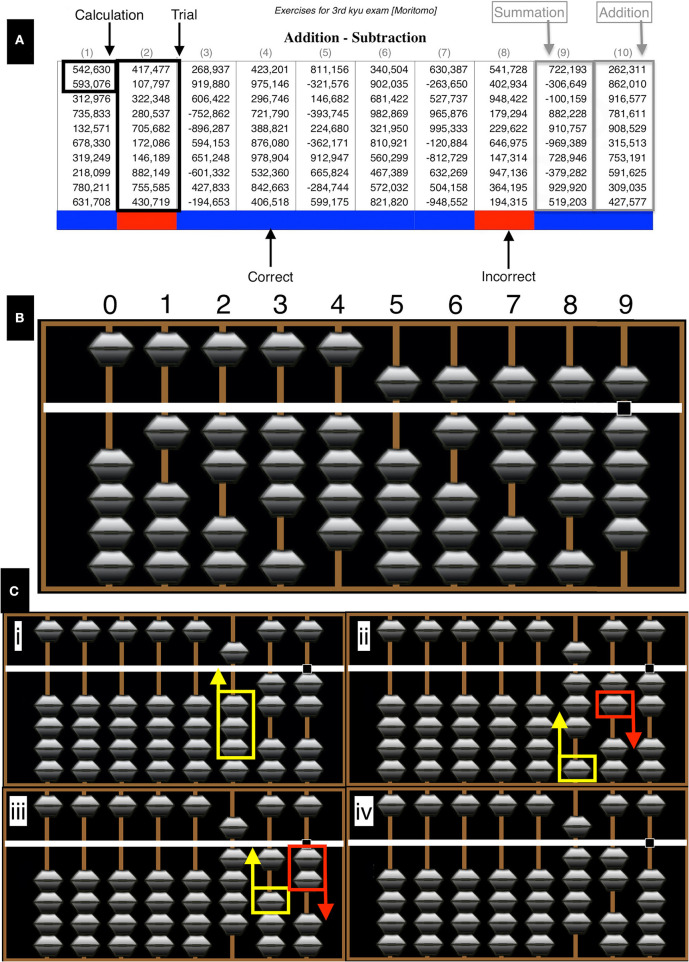
Test example. **(A)** The test consisted of 10 columns (trials) of 6-digit numbers labeled 1–10. There were six addition and four summation trials per test sheet. To provide a visual indicator of performance in each sheet, color coding at the last cell of each column, where a blue or red color was used to indicate a correct or incorrect result, respectively. **(B)** Digits from 0 to 9 are represented on each rod by adding the numerical value of all beads contacting the central horizontal beam. The lower beads have a numerical value of 1, whereas the single upper bead has a value of 5. **(C)** An example of an addition operation (522+398) showing the principles of number representation and complementary number calculations (details provided in the text). Images generated with abacus software http://www.komodousa.com.

The learning period duration was 60 days, followed by 8 test days to assess the model forecasts. Tests were conducted between 7:00 and 7:30 a.m. Test sheets were randomly generated from the Soroban exam website (www.sorobanexam.org). Each sheet lays out both the questions and answers of a set of 10 columns of numbers (called a trial in this study). A sheet was composed of a mixture of six addition and four summation trials. The test difficulty was set to what is known in the Japanese Soroban exam system as difficulty level 3rd kyu, which consists of numbers ranging between 100,000 and 999,999. At the end of the test, a trial outcome was compared with the printed result and recorded. The last cell of the trial column was color coded with either a blue or red color, to indicate a successful or unsuccessful trial, respectively. Also, the time to complete a set of additions or summations was recorded in seconds. An example test sheet is provided in Figure 1. The in-built iOS voice over app (High Sierra 10.13.6) was used to vocalize the list of numbers from a .pdf list from the test sheet. Cumulative calculation time was defined as calculation time in seconds for adding 10 sequential 6-digit numbers, which either represented the addition task only (CT_Add_) or a combination of addition and subtraction (CT_Sum_). The author had limited prior experience with the Soroban (self-taught in 2017). Refer to the Supplementary Material section for the learning and test phase of the dataset.

### 2.2. The Soroban

The Soroban is a mechanical calculator, of which the origins are traced back to Mesopotamia, 2,500 years BC. Basic mathematical operations (addition, subtraction, multiplication, and division) can be performed using the device. There are two principles of operation: all calculations are performed as a number is pronounced, i.e., from the left to right. In addition, it reduces the complex mental mathematics to a simpler task, by using an algebraic principle of the method of complements, being in this case, either five or ten (Association, 1989; Schumer, 1999). Number representation and an example calculation is demonstrated in Figure 1. For clarification colored arrows are the next move in an operation (yellow = up, red = down). All computations are performed from left to right. Beads in contact with the central horizontal beam are considered in the final calculation. In this example of an addition operation (522+398), computation is started by representing the number 522 on the Soroban (Figure 1Ci). The number (300) is then added to the hundreds rod (Figure 1Cii). Direct representation can take place with this step as there are an adequate number of beads not contacting the central beam. Adding 90 to the tens rod is not possible directly therefore, the complementary technique is used, in this method 100 − 10 = 90, a bead is added to the hundreds rod and another subtracted from the tens rod (Figure 1Ciii). To add 8 to the ones rod the complementary technique again, where 10 − 2 = 8, a bead is added to the tens rod and 2 subtracted from the ones rod giving a result of 920 (Figure 1Civ).

#### 2.2.1. Time Series Model Description

An ARIMA time series model is mainly defined by three terms (p,d,q), which represent the autoregressive (p), integrative (d), and the moving average (q) parameters of the model, respectively. The general mathematical description of the model is provided below (Box et al., 2015):

(2) $$\begin{array}{l} \varphi \left(B\right)z_{t}=\phi \left(B\right){▽}^{d}z_{t}=\theta_{0}+\theta \left(B\right)a_{t} \end{array}$$

where

(3) $$\begin{array}{l} \phi \left(B\right)=1-\phi_{1}\left(B\right)-\phi_{2}{\left(B\right)}^{2}\ldots .-\phi_{p}{\left(B\right)}^{p} \end{array}$$

(4) $$\begin{array}{l} \theta \left(B\right)=1-\theta_{1}\left(B\right)-\theta_{2}{\left(B\right)}^{2}\ldots .-\theta_{q}{\left(B\right)}^{q} \end{array}$$

(B) is the backward shift (lag) operator, which is defined by $B^{k}z_{t}$ = *z*_*t* − *k*_. This operator is convenient for describing the process of shifting between successive points in the series. That is to say B, operating on *z*_*t*_, has the effect of shifting the data back one period.
ϕ(*B*) is the autoregressive polynomial operator in B of degree (p); it is assumed to be stationary, that is, the roots of ϕ(*B*) = 0 lie outside the unit circle.
φ(*B*) = θ(*B*)▿^*d*^ is the generalized autoregressive (backward difference ▿*z*_*t*_) operator; which is a non-stationary operator with d of the roots of φ(*B*) = 0 equal to unity, that is, d unit roots. The backwards difference operator is defined as ▿*z*_*t*_=*z*_*t*_ − *z*_(*t*−1)_=(1 − *B*)*z*_*t*_. Differencing is used to stabilize the series when the stationarity assumption is not met.
θ(*B*) is the moving average polynomial operator in B of degree (q); it is assumed to be invertible, that is, the roots of θ(*B*) = 0 lie outside the unit circle.
The error term (*a*_*t*_), which is assumed to have a Gaussian distribution, with a mean (μ) of zero and a constant variance of $\left(\sigma_{\epsilon }^{2}\right)$.

In practical terms, fitting the ARIMA model requires defining the model order (p,d,q). The autoregressive (ar) term, determines the value of (p), which is a datapoint in the series weighted by the value of proceeding data points. The term is given a number (ar_n_); this represents the lag value in the series from where the correlation was calculated. The moving average (ma_n_) corrects future forecasts based on errors made on recent forecasts; this term determines the (q) of the model order calculated from the partial autocorrelation function, which is a summary of the relationship between an observation in a time series with observations at prior time steps with the relationships of intervening observations removed. The integrated (d) portion of ARIMA models does not add predictors to the forecasting equation, rather, it indicates the order of differencing that has been applied to the time series to remove any trend in the data and render it stationary.

#### 2.2.2. Statistical Analysis

Data was analyzed in R. The distributions of CT_Add_ and CT_Sum_ were modeled using the fitdistplus() package (Delignette-Muller and Dutang, 2015). Results are expressed as the median, range and interquartile range (IQR). Pearson's Chi-squared test (χ^2^) with Yates' continuity correction was used to assess the differences in the accuracy of the calculated result between the addition and summation tasks. The Wilcoxon ranked sum test was used to assess the differences in CT_Add_ and CT_Sum_.

Parameters of Wright's model were estimated using the learningCurve() package in R. This package uses Equation (1) in its calculations (Boehmke and Freels, 2017). The learning natural slope estimate (b) was calculated using the equation:

(5) $$\begin{array}{l} b<-\frac{Log_{10}T-Log_{10}t}{Log_{10}n-1} \end{array}$$

where T = total time (or cost) required to produce the first n units, t = time of all trials, *n* = total trials. The learning rate estimate (s) is calculated from the natural slope estimate by applying the following equation:

(6) $$\begin{array}{l} s=\frac{10^{b*log10\left(2\right)+2}}{100} \end{array}$$

To forecast the 100th additional trial direct substitution in Equation (1) of (x) was done, where x = time for the 947th and 663rd attempt for the addition and summation tasks, respectively (a = the time for the first attempt in each of these tasks).

Univariate linear regression was utilized to assess the correlation between the time to perform the task and the number of trials and the equation of the best line fit was derived. The adjusted correlation coefficient (*R*^2^) was used to represent the proportion of the variance explained by the model fits. The residual standard error (RSE) was used to assess model fit to the residuals.

The autoregressive integrated moving average model (ARIMA) was used for forecasting. The time series was plotted together with autocorrelation (acf) and partial autocorrelation functions (pacf). Although automated fitting of the time series (auto.arima) from the forecast package was initially used, acf and pacf graphs were used to confirm the order (p,q,d) of the series. After visual inspection of the time series plot suggested stationarity (mean, variance, and auto-covariance being independent from time), this assumption was confirmed by applying two statistical tests: the augmented Dickey-Fuller test (ADF), which is unit root test for stationarity, and The Kwiatkowski–Phillips–Schmidt–Shin test (KPSS). Unit roots (difference stationary process, i.e., a stochastic trend in a time series, sometimes called a “random walk with drift”), which exist in a time series if the value of α=1 in the general time series equation:

(7) $$\begin{array}{l} Y_{t}=\alpha Y_{t-1}+\beta X_{e}+\epsilon \end{array}$$

The lag length (k) was chosen for this test (CT_Add_ k = 6, and CT_Sum_ k = 5) to avoid serial correlation of the residuals by choosing the last statistically significant lag, as determined by the partial autocorrelation function (pacf). The KPSS test was then applied, which is used for testing the null hypothesis that an observable time series is stationary around a deterministic trend (mean) or is non-stationary due to a unit root. Selection of the ARIMA model order (p,d,q) was chosen using the automated R auto.arima() command, which combines unit root tests, minimization of the corrected Akaike's Information Criterion (AICc) and Maximum likelihood estimation (MLE) to obtain an ARIMA model (Hyndman and Athanasopoulos, 2018). Validity of the model parameter choice was confirmed by plotting the autocorrelation (ACF) and partial autocorrelation (PACF) plots of the stationary data to determine a possible model candidate as suggested by the minimal AICc. Model fitting diagnostics also considered the lowest root mean square error (RMSE) and mean absolute percentage error (MAPE). A plot of the ACF of the residuals was done to confirm if the residuals appeared to be white noise. Once these criteria were met the forecast equations were calculated. The characteristic roots of both time series equations were plotted to assess whether the model is close to invertibility or stationarity in relation to the complex unit circle. Any roots close to the unit circle may be numerically unstable, and the corresponding model will not be suitable for forecasting. This possibility is mitigated through the auto.arima() function, which avoids selecting a model with roots close to the unit circle (Hyndman and Khandakar, 2008). Plotting the fitted model against the time series plot was performed. The models were tested for autoregressive conditional heteroscedasticity using the McLeod-Li test. Plotting the acf of the residuals and the Ljung-Box test were performed to assess for autocorrelations. In order to assess the model performance, the mean point forecast was reported from each model. In addition, forecasted data was generated from the model parameters and compared with actual test performance for an additional 100 trials using a pairwise-Wilcoxon test with Bonferroni correction. A *p*-value of < 0.05 was considered statistically significant for all tests.

## 3. Results

**Figure 2:**
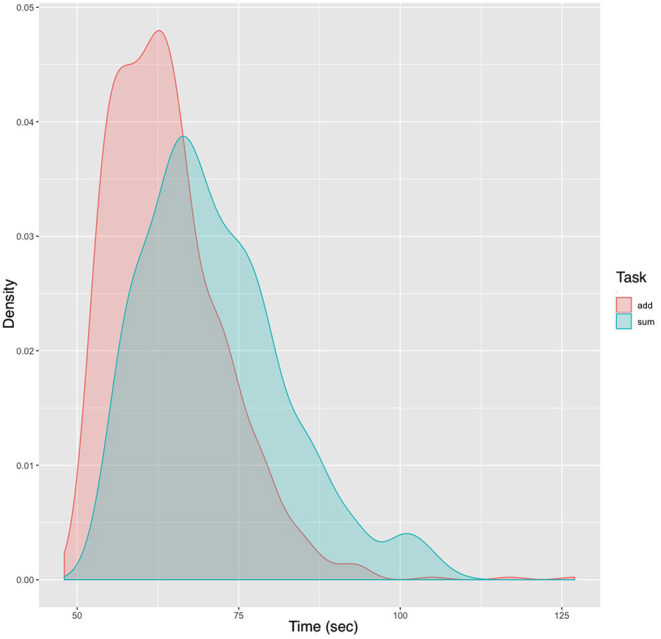
Density plot of performance subset by task. The density plot uses kernel smoothing to reduce noise in the data, which generates a more defined distribution. The peaks of a density plot display represents values that are concentrated over the test interval. Noted in the plot are the narrower range of values and the shift to the left of the peak density for CT_Add_ compared to CT_Sum_.

Over 60 days a total of 1,410 trials were conducted. The actual test time was 26.28 h during which a total of 847 addition and 563 summation trials were conducted. A variable number of trials, ranging from 0 to 70 trials per day were carried out. The distribution of both CT_Add_ and CT_Sum_ was non-normal and best fit a skewed exponential power type 4 distribution (model coefficients fit *p* < 0.0001). The skew and kurtosis of CT_Add_ were 1.38 and 7.46, respectively, whereas the corresponding values for CT_Sum_ were 0.80 and 3.40. The density distribution plot is demonstrated in Figure 2.

**Figure 3:**
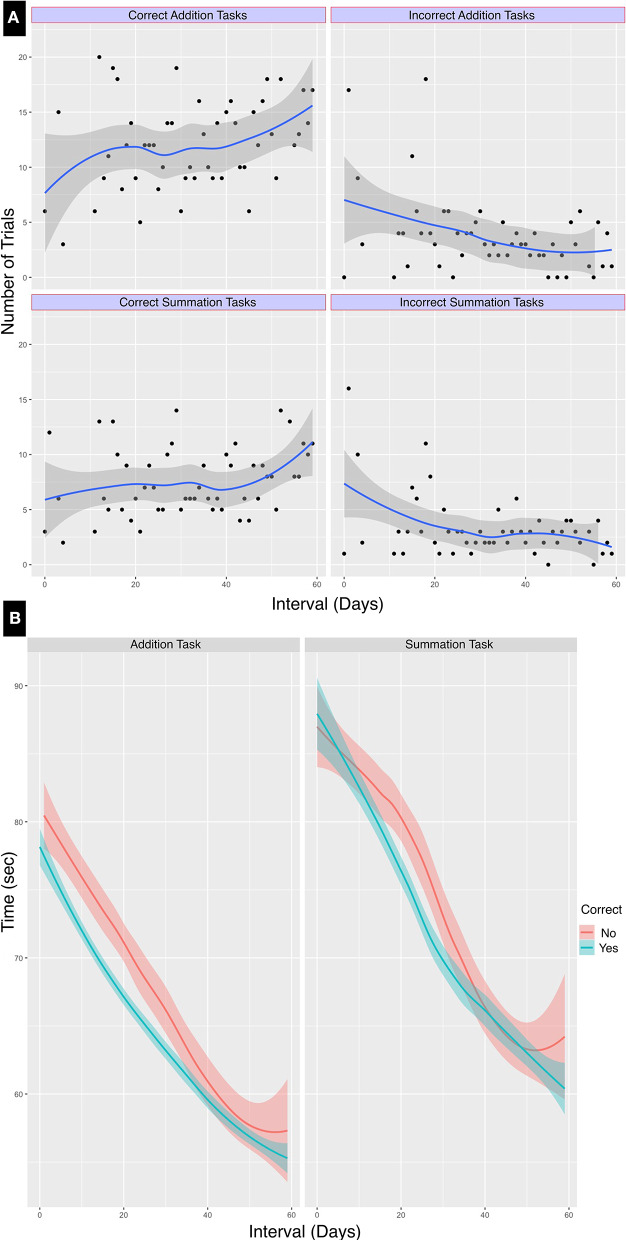
Line graph of performance classified by task type and duration. Loess smoothed line graphs with 95% confidence intervals show the progress in test performance classified by the calculation result accuracy over the test interval of 60 days. There was both **(A)** an increase in the number of correct calculations and **(B)** a reduction of test time for addition and summation tasks over the learning period.

Addition tasks, being the simpler of the two, were more likely to yield an accurate result, and this difference compared to the outcome of the summation task was statistically significant (χ^2^ = 9.33, df = 1, *p* < 0.002). There was an increasing trend of total successful trials, as demonstrated in Figure 3. As expected, there was an improved performance with training, there were correct outcomes were recorded for 449/660 (68%) of trials in the first half compared to 598/750 (79.7%) in the second half of the learning period. The outcome of all tests classified by task type are summarized in Table 1.

**Table 1:** Learning outcome performance.

**Trial**	**Correct**	**Incorrect**	**Total**
**LEARNING PERIOD (DAY 0–59)**			
Addition	654 (r = 77%, c = 62%)	193 (r = 23%, c = 53%)	847 (60%)
Summation	393 (r = 70%, c = 38%)	170(r = 30%, c = 47%)	563 (40%)
Total	1047 (74%)	363 (26%)	1410
**TESTING PERIOD (DAY 0–7)**			
Addition	82 (r = 82%, c = 51.9%)	18 (r = 18.0%, c = 42.9%)	100 (50%)
Summation	76 (r = 76%, c = 48.1%)	24 (r = 24%, c = 57.1%)	100 (50%)
Total	158 (79%)	42 (21%)	

*A comparison of the outcome of the trials during the learning and test periods. The learning period (60 days). The testing period (8 days) was to verify the accuracy of the model predictions, r = percentage by row, c = percentage by column*.

Table 2 Summarizes the performance timing characteristics. From this table it can be noted that the median CT_Add_ was shorter compared to the median CT_Sum_, this difference persisted throughout the learning period duration. Trials that concluded with an accurate calculated result (median time = 64 s, IQR = 13, range 48–117 s) were quicker compared to those where the result was incorrect (median time = 70 s, IQR = 15, range 51–127 s). These differences between addition, summation and performance times at the mid-learning interval were statistically significant (*p* < 0.0001). These results are shown in Figure 3.

**Table 2:** The time to complete the tasks showed an expected reduction with learning.

			**Range**	
	**Median**	**IQR**	**Min**	**Max**
**TOTAL LEARNING PERIOD**				
CT_Add_	63	12	48	127
CT_Sum_	70	14	53	108
**INITIAL LEARNING PERIOD (DAY 0–29)**				
CT_Add_	68	10	57	127
CT_Sum_	78	14	59	108
**LATE LEARNING PERIOD (DAY 30–59)**				
CT_Add_	58	7	48	88
CT_Sum_	64	9	53	87
**TEST PERIOD**				
CT_Add_	53	4	45	69
CT_Sum_	58	6.25	47	75

*The learning period was 60 days during which 1,410 trials were conducted, the performance modeled, and forecasting equations were used to generated expected timing data. The test period consisted of 100 additional trials*.

### 3.1. Mathematical Models

#### 3.1.1. Wright's Model

The time for the first event for CT_Add_ was 76 s, the total time 54,302 s and the number of trials 847, whereas for CT_Sum_ the first event was 74 s, the total time 40,297 s and the number of trials was 563. The learning rate was 0.98 and 0.99 for the addition and the summation tasks, respectively, this was calculated as a ratio of learning time at each doubling of the event i.e., time to event 1/time for event 2, time for event 2/time for event 4, time for event 4/time for event 8……etc. The natural slope is calculated by dividing the log of the learning rate by log2, this was further refined by calculating the natural slope estimate when the total number of trials, total time of all trials and the time for the first trial is known. Substituting in Equation (5), the natural slope estimate was −0.025 and −0.005 for the addition and summation tasks, respectively. The learning rate was further refined by taking into account the natural slope estimate by applying Equation (7), therefore the learning rate was estimated at 0.983 for the addition task and 0.996 for the summation task.

**Figure 4:**
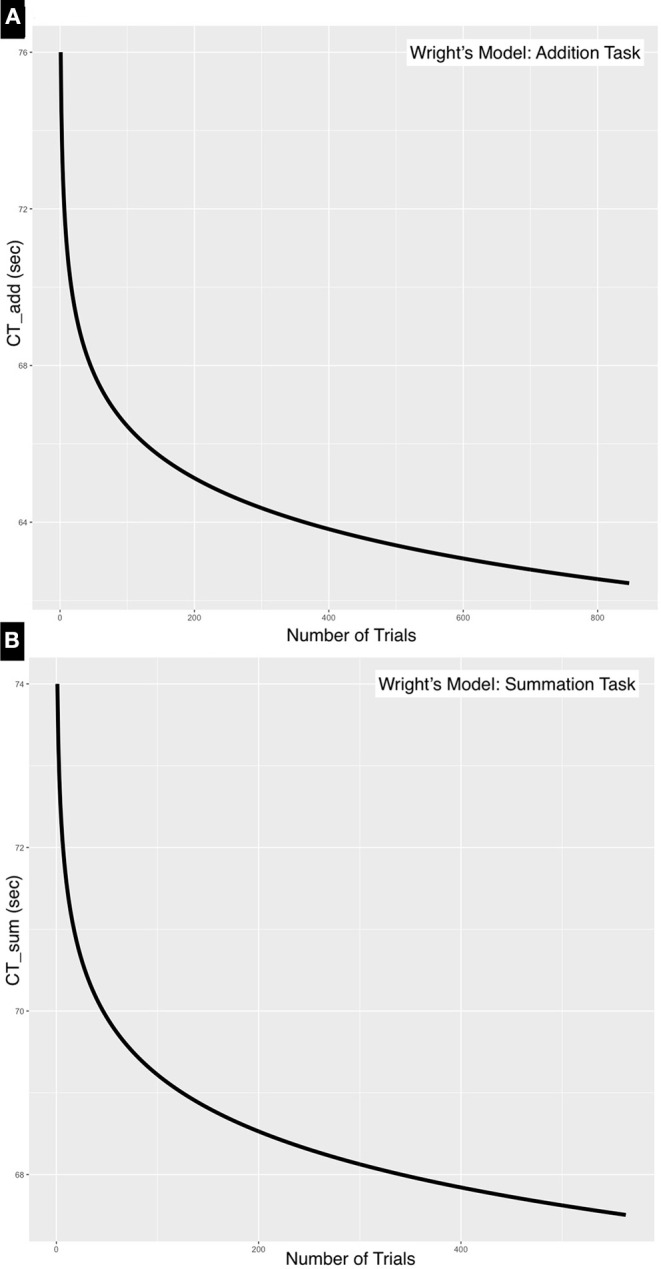
Negative exponential curves for **(A)** addition and **(B)** summation tasks. These learning curves were generated using Wright's model. The natural slope estimate is steeper for CT_Add_ (−0.025) compared to that of CT_Sum_ (−0.005). This is a consequence of the simpler addition task reaching a plateau quicker than the more complex summation task.

Substituting in Equation (1) where (x) is the forecasted performance time at the end of the 100th trial, CT_Add_ would be 62.24 s and CT_Sum_ would be 67.34 s. The plot of the model parameters is outlined in Figure 4.

#### 3.1.2. Univariate Linear Regression

In the simplest case, when the variance in performance was disregarded, univariate linear regression was used to estimate the correlation between the cumulative time to perform both tasks and number of trials conducted. Consequently, the following equations were derived, where (x) is a variable representing the number of trials:

(8) $$\begin{array}{l} CT_{Add}=-0.027\left(\pm 0.00085\right)\cdot x+75.63\left(\pm 0.41\right) \end{array}$$

(9) $$\begin{array}{l} CT_{Sum}=-0.051\left(\pm 0.0021\right)\cdot x+85.63\left(\pm 0.64\right) \end{array}$$

**Figure 5:**
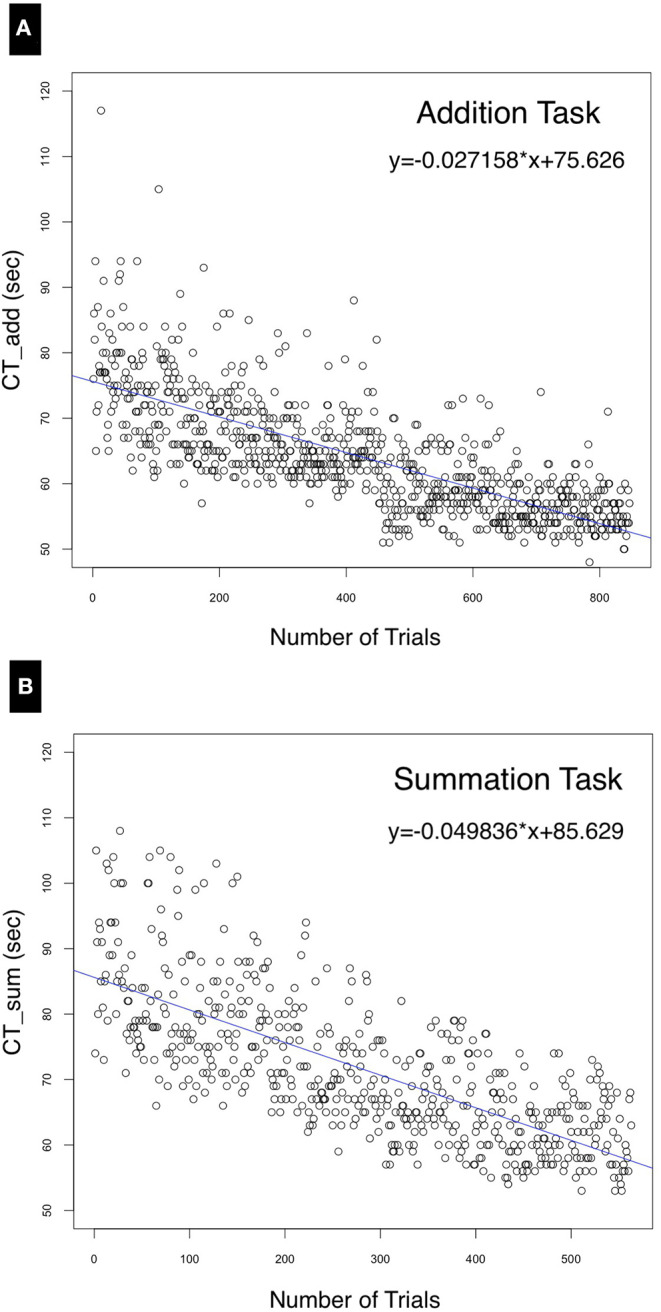
Univariate linear regression model. correlating the performance time (seconds) with the number of trials for **(A)** the addition and **(B)** the summation trials. The distribution of residuals from the regression line for **(B)** is further from the regression line compared to **(A)** due to the wider variance in performance time for the summation task.

The intercepts of Equations (8) and (9), +75.63(±0.41) and +85.63(±0.64), respectively, indicate the values of CT_Add_ and CT_Sum_ at baseline (day 0) when commencing the test, and therefore represent the level of prior expertise with the task. Both the negative slope of the regression line (Figure 5) and the negative (x) variable coefficient demonstrate a reduction of performance time with learning. The summation task resulted in a higher RSE (7.55, df = 561) compared to the addition task (6.02, df = 845) due to the lower deviation from the regression line as shown by the median of the residuals for summation (−1.46) compared to (−0.6) for the addition task. The model's predictor (number of trials) explained about half of the variance in the dependent variable (CT_Add_ and CT_Sum_) as indicated by an adjusted *R*^2^ of 0.55 and 0.54 for Equations (8) and (9), respectively. Statistical significance was achieved for all model coefficients (*p* < 0.0001). Substituting in these equations, the forecast for the for the 100th additional forecasted trial yields a mean time of CT_Add_ = 50.06 s and CT_Sum_ = 51.82 s.

#### 3.1.3. Autoregressive Integrated Moving Average Model (ARIMA)

**Figure 6:**
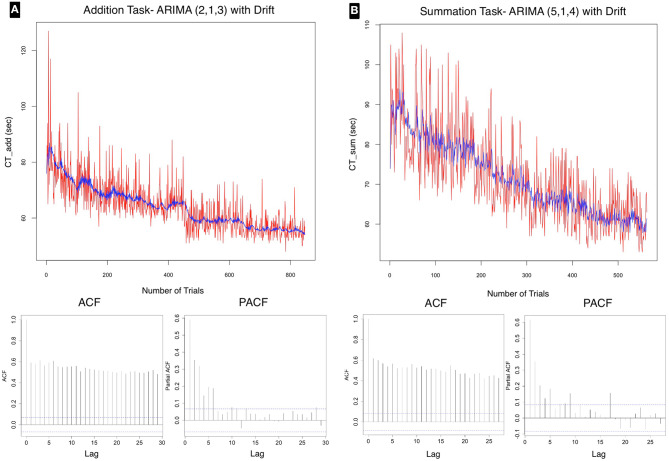
Time series plot for **(A)** addition and **(B)** summation tasks with autocorrelation (acf) and partial autocorrelation (pacf) correlograms, which allow visual interpretation of the correlation of present data points with past points in the series. The time series for both tasks is demonstrating a declining trend. The acf plots for both tasks show a geometric pattern, with strong correlation of the sequential points up to lag 30. The pacf showed significant correlation at the 95% confidence interval up to lag 6 for addition, and lag 5 for the summation task. These tests confirm a gradual improvement in performance over the test time with a <20% correlation of test scores at each 6th trial in the series for addition and 5th trial for summation at the 95% confidence interval. A favorable model fit in the time series is demonstrated as a blue line in each of the series plots.

The time series was of sub-daily frequency ranging from 10 to 70 trials (median 30) per day. There were three discontinuities in testing: interval 2, intervals 5–10, and interval 53. The interval was calculated from day 0 at the commencement of the test. As shown in Figure 6 there was an overall declining average for both the addition and the summation trials. The ADF test confirmed stationarity of the series for both CT_Add_ (ADF value = −6.86, *p* < 0.01) and CT_Sum_ (ADF value = −6.86, *p* < 0.01). However the KPSS test was statistically significant for both CT_Add_ (KPSS value = 10.23, *p* < 0.01) and CT_Sum_ (KPSS value = 6.81, *p* < 0.01), this result indicated that the time series had stationary autoregressive terms (ar) and non-stationary moving average terms (ma), which was consistent with the declining trend in the performance time for both tasks as shown in Figure 3, this analysis confirmed that the series was weakly stationary and that differencing using the ARIMA model was required to render the series stationary for further analysis.

The correlograms in Figure 6 show, for both CT_Add_ and CT_Sum_ that the acf is highly correlated at all lag values up to lag 30; therefore the suggested q would be of order 1. The pacf plot is used to select the order of the p term. For the addition task the highest significant value was at lag 6, whereas the value for the summation task was at lag 5. Therefore, a custom ARIMA model would be (6, 1, 1), AIC = 5409.09 for addition and (5, 1, 1), AIC = 3863.65 for the summation task.

Software packages (like R) provide the option of an automated ARIMA model order approximation, when this was trialed for the series, an 18 and a 23-order permutation was tested for addition and summation, from both these approaches the model order (2, 1, 3) with drift for addition, where the AIC was 5403.13 and the order for summation was (5, 1, 4) with drift, where the AIC was 3852.61. Hence the automated approximation provided more favorable model parameters. The coefficients and accuracy criterion of the model are listed in Table 3.

**Table 3:** ARIMA model parameters.

		**ar1**	**ar2**		**ma1**	**ma2**		**ma3**	**Drift**	
**ADDITION TRIALS ARIMA (2,1,3) MODEL PARAMETERS**										
Coefficients		−1.3976	−0.864		0.4623	−0.4916		−0.7876	−0.0321	
se		0.0922	0.0763		0.1077	0.0936		0.0766	0.0115	
*p*-value		<0.0001	<0.0001		<0.0001	<0.0001		<0.0001	<0.005	
RMSE		5.84								
MAPE		6.32								
AIC = 5403.13, AICc = 5403.27, BIC = 5436.32										
	**ar1**	**ar2**	**ar3**	**ar4**	**ar5**	**ma1**	**ma2**	**ma3**	**ma4**	**Drift**
**SUMMATION TRIALS ARIMA (5,1,4) MODEL PARAMETERS**										
Coefficients	−1.8077	−1.5259	−0.4061	0.2994	0.0916	0.9703	−0.0115	−0.9706	−0.8742	−0.0495
se	0.0859	0.1229	0.1278	0.099	0.0483	0.0749	0.0426	0.0518	0.0591	0.0089
*p*-value	<0.0001	<0.0001	<0.001	<0.002	<0.06	<0.0001	<0.8	<0.0001	<0.0001	<0.0001
RMSE	7.28									
MAPE	8.02									
AIC = 3852.61, AICc = 3853.09, BIC = 3900.26										

*ARIMA model parameters for the addition and the summation tasks. ar, autoregressive coefficients; ma, moving average coefficients; se, standard error. RMSE, root mean square error; MAPE, mean absolute percentage error; AIC, akaike information criterion; AICc, corrected akaike information criterion; BIC, Bayseian information criterion*.

Autoregressive conditional heteroscedasticity (ARCH) among the lags was considered, the Mcleod-Li test for the addition model ARCH effects are were absent, for the summation task, from a total of 30 lags there was minimal (13%) ARCH effects in lags 3–7.

The following equations can be used to describe the time series fitted in Figure 6 derived in standard notation:

(10) $$\begin{array}{c} CT_{Add}\left(Y_{t}\right)=-1.39Y_{\left(t-1\right)}-0.86Y_{\left(t-2\right)}+0.46e_{\left(t-1\right)}-0.49e_{\left(t-2\right)}\\ \;-0.79e_{\left(t-3\right)}-0.032e_{t} \end{array}$$

(11) $$\begin{array}{l} CT_{Sum}\left(Y_{t}\right)=-1.81Y_{\left(t-1\right)}-1.53Y_{\left(t-2\right)}-0.41Y_{\left(t-3\right)}\\ \;+0.31Y_{t}\left(t-4\right)+0.092Y_{\left(t-5\right)}+0.97e_{\left(t-1\right)}\\ \;-0.01e_{\left(t-2\right)}-0.97e_{\left(t-3\right)}-0.87e_{\left(t-4\right)}-0.051e_{t} \end{array}$$

Where (Y) is the autoregressive term, (e) is the moving average term, (e_t_) is the error term and (t-n) is the lag (time interval between two data points).

**Figure 7:**
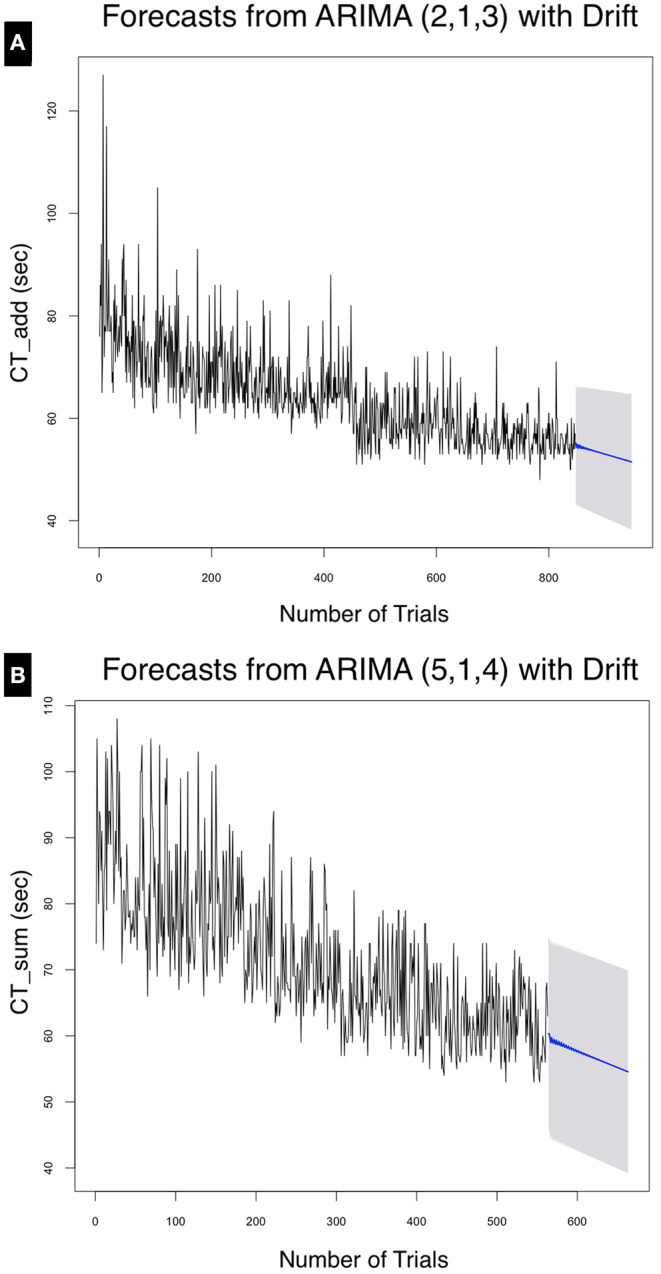
Forecasts for **(A)** CT_Add_ and **(B)** CT_Sum_ at the 95% confidence interval for the next 100 trials. The point forecast for the 947th trial was calculated at 51.51 ± 13.21 s, whereas that for CT_Sum_ at the 663rd trial was calculated at 54.57 ± 15.36 s.

As shown in Figure 7. The mean forecasted performance for the 100th trial for CT_Add_ was 51.50 ± 13.21 and for CT_Sum_ was 54.57 ± 15.37. From Table 3 using (MAPE) to measure forecast accuracy, the model was able to forecast with an error of 6.42% for the addition task and 8.02% for the summation task.

Independence of the residuals for both ARIMA models was evaluated using the acf plot of the residuals, which showed the absence of autocorrelation. This was confirmed by the Ljung-Box test. Parameters for the addition task (χ^2^ = 759.99, df = 800, *p*-value = 0.84) and the summation task (χ^2^ = 480.93, df = 500, *p*-value = 0.72) failed to achieve statistical significance, therefore an absence of serial autocorrelation in both series, thereby confirming an appropriate model fit. The three model comparisons on predicting the actual means on repeating the tests for a further 100 trials for each of the addition and the summation tasks are listed in Table 4.

**Table 4:** Mean point forecast.

	**CT_Add_**	**CT_Sum_**
POINT FORECAST COMPARISON (SEC)^*^		
Wright's model	62.24 (+9.38)	67.34 (+8.36)
Linear regression	50.06 (−2.74)	51.82 (−7.16)
ARIMA	51.50 (−1.36)	54.57 (−4.41)
Actual mean	52.86	58.98

*Comparison of the mean point forecasted value at the 100th trial for CT_Add_ and CT_Sum_*.

* *The number in brackets is the difference from the actual mean in seconds. The ARIMA model provided the closest prediction to actual performance*.

The forecasted mean ARIMA model values offered a closer match with actual test performance (*p*-value CT_Add_ = 1.0, CT_Sum_ = 0.054), this in contrast to both Wright's model and univariate linear regression, for which mean values differed from these test (*p*-value < 0.0001) for both tasks. Simulated data for the three models for the forecasted period were compared using the paired Wilcoxon rank sum test, which showed no difference for the ARIMA model values from the actual test values (*p*-value CT_Add_ 1.0, CT_Sum_=0.054), this is in contrast to both Wright's and the linear regression models which forecasted statistically significant (<0.0001) values for both tasks.

## 4. Discussion

The ARIMA model provided a more accurate approximation to actual performance after 100 additional trials, compared to both univariate linear regression and Wright's model. Considering the model means, in their predictions, the former overestimated and the latter underestimated the actual performance (Table 1). Many formal models of learning generate smooth learning curves, which are seldom observed except at the level of average data (Glautier, 2013). In this example both Wright's model and simple linear regression hide important information regarding performance variance. The ARIMA model predicted CT_Add_ more accurately than that of CT_Sum_, and this may be accounted for by the larger number of addition trials of which the test trials constituted 60% compared to the more complex summation task, where the test trials constituted approximately 40% of the total learning dataset. As shown in Figure 2 the distribution of calculation times for both tasks were non-linear. In addition, the decline in both CT_Add_ and CT_Sum_ followed a non-linear trajectory over time (Figure 3). These patterns are consistent with the three phases of learning theory, which predict a three phase life cycle: the incipient phase during which a familiarization with the task occurs, which is characterized by a slow improvement; the learning phase, is where most of the improvement takes place; and the final phase, where the performance levels off (Carlson and Rowe, 1976). Whereas, prior knowledge of the task would have masked the incipient phase, the limitations of the univariate linear model become apparent by concealing the different performance phases altogether due to the constant slope of its regression line.

The neurophysiological basis of the Soroban remains unclear, however, it is known that computations using the Soroban involves a higher level of visual imagery (Tanaka et al., 2012). A longitudinal functional magnetic resonance imaging study of a patient with abacus-based acalculia suggested an important role in the parietal cortex and the dorsal premotor cortex in arithmetic ability of abacus users (Tanaka et al., 2012). Several cognitive processes required for mental arithmetic take place in these regions including retrieval, computation, reasoning, and decision making about arithmetic relations in addition to resolving interference between multiple competing solutions (interference resolution) (Menon, 2010). These factors may have played a role in the differences in variance in the performance of tasks as shown in Figures 4–6.

Calculation time for both addition and summation tasks as shown in Figure 6 demonstrate a predictable downward trend and a slightly higher learning rate. The slope in the univariate linear regression was more negative for CT_Sum_ than CT_Add_, although the former was a more complex task. There may be some influence of the difference in the scale of comparison, as the number of trials for the summation tasks were less than the addition tasks by about 20%. A comparison of the ARIMA model pacf plot in Figure 6 also suggests a slightly higher learning rate with summation compared to the addition task as indicated by the loss of correlation over shorter lags with the former task. In Wright's model the steeper natural slope estimate was approximately twice that for addition compared to summation, perhaps reflecting the higher complexity of the latter and a more gradual departure from the learning phase. As multiple neural systems and pathways involved in mathematical information processing mainly the parietal cortex, prefrontal cortex with several functional dissociations between brain regions differentially involved in specific operations such as addition, subtraction, and multiplication have been suggested in literature. Menon (2010), it is therefore difficult to speculate on the underlying structural reason behind the detected difference in learning speed. The other possibility behind the large variance seen in summation tasks is the measured difference in the number of complementary calculations per computation in each trial which was not considered in this experiment.

Inherent to the mathematical property of a times series analysis, is the capability of the model to capturing both linear and nonlinear relationships of the variables in the model. This property distinguishes it from other analysis methodologies which are either linear or non-linear (Yanovitzky and VanLear, 2008). In addition to describing the learning process in rigorous mathematical terms at an individual level, a psychological benefit is conferred to the test subject through accurate feedback of the improvement in performance. The limitations of this technique include the amount of data required to perform the analysis and the mathematical skill required to interpret the results. The protracted nature of the data collection requires a commitment in the testing process and may hinder some practicality as a routine test of learning performance. Although with the current experiment, the model fit was appropriate and delivered a high level of forecasting accuracy, most time series model predictions falter with extended forecast times due to non-stationarity, cohort effects, time-in-sample bias, and other challenges of longitudinal analyses (Taris, 2000; Yanovitzky and VanLear, 2008). Therefore, it is not clear from the current analysis how far into the future the forecast would be able to extend and retain its predictive accuracy. While the Soroban is still widely taught in Asian schools and therefore time series modeling may be beneficial for a more directed approach to teaching this skill, it may not apply to a wider population in other regions of the world where the use of the Soroban is less common. Future studies involving simultaneously recording an encephalogram may uncover wave activity associated with performance and the neurological basis of calculation errors in this task.

## 5. Conclusion

Time series analysis, by capturing the variance in performance may offer a more accurate mathematical representation of the learning process than classical learning theory models. The additional advantage of the ARIMA model to accurately forecast cognitive performance, with an accuracy exceeding that of both Wright's model and univariate linear regression, offers a potential for a wider applications for evaluation of cognitive function.

## Data Availability Statement

All datasets generated for this study are included in the article/Supplementary Material.

## Ethics Statement

Ethical review and approval was not required for the study on human participants in accordance with the local legislation and institutional requirements. The patients/participants provided their written informed consent to participate in this study. Written informed consent was obtained from the individual(s) for the publication of any potentially identifiable images or data included in this article.

## Author Contributions

AA-R designed the study, analyzed the data, and created the current version of the manuscript.

## Conflict of Interest

The author declares that the research was conducted in the absence of any commercial or financial relationships that could be construed as a potential conflict of interest.

## Supplementary Material

Data Sheet 1 Learning period addition and summation times.

Data Sheet 2 Testing period addition and summation times. For both Supplementary Data Sheets: rate, iOS accessibility program voice over speed; kyu, Calculation difficulty rate; Correct, correct answer calculated; time, test time in seconds; interval, Duration in days from start to the end of the learning period.
